# Supplementary material for: Resting-State fMRI Dynamics and Null Models: Perspectives, Sampling Variability, and Simulations
Source: Front Neurosci. 2018 Sep 6;12:551. doi: 10.3389/fnins.2018.00551 (PMC6135983; doi:10.3389/fnins.2018.00551)
Supplement: Supplementary file 1 [file Data_Sheet_1.DOCX]

**Technical Supplement**

**SCC Multivariate Gaussian Processes**

Let $w_{m,T}^{(i)}$ be one $m\times T$ matrix of white Gaussian noise, $\boldsymbol{P}$ the average power spectrum for all subjects and all $m$ network TCs in an actual resting state fMRI study, and $\boldsymbol{C}_{m}$ the $m\times m$ population mean cross-network covariance. The set of timeseries one gets by inverse Fourier transforming a set of $T$ normalized random complex coefficients $\theta_{i}=\frac{\omega_{i}}{\left| |\omega_{i} \right||}, i=1,2,\ldots,T$ weighted according to a fixed template spectrum $\boldsymbol{P}=\left\{ p_{1},p_{2},\ldots,p_{T} \right\}$, ie, the set:

$$S\left( \boldsymbol{P,}T \right)\boldsymbol{=}\left\{ s\left( t \right):s\left( t \right)=iFFT\left( \left\{ p_{1}\theta_{1},p_{2}\theta_{2},\ldots,p_{T}\theta_{T} \right\}, \mathrm{random} \vec{\boldsymbol{\theta}}. \mathrm{fixed} \boldsymbol{P} \right) \right\}$$

includes the discrete analogue (as we are working with computers on finite samples) of all length-$T$ piecewise continuous functions with spectrum $\boldsymbol{P}$. In particular, these timeseries exhaustively exhibit the full range of epochal behavior possible under the global spectral constraint $\boldsymbol{P}$. Since $\boldsymbol{P}$ is the average spectrum of network TCs from a real rs-fMRI study, this ensures that the epochal signatures of CASE-driven rs-FRBD are replicated in $S\left( \boldsymbol{P,}T \right)$. The set of $m\times T$ multivariate timeseries

$$S_{m}\left( \boldsymbol{P,}T \right)\boldsymbol{=}\left\{ \boldsymbol{s}\left( t \right)=[s_{1}\left( t \right);s_{2}\left( t \right);\ldots;s_{m}\left( t \right)]:s_{i}\left( t \right)\in S\left( \boldsymbol{P,}T \right)\boldsymbol{,}i=1,2,\ldots,m \right\}$$

similarly exhaust the range of *m*-fold jointly realizable within-timeseries epochs subject to the duration $T$ and spectral constraint $\boldsymbol{P}$. Finally, the set

$$S\left( \boldsymbol{P,}T,\boldsymbol{C}_{m} \right)\boldsymbol{=}\left\{ \boldsymbol{s}\left( t,\boldsymbol{C}_{m} \right)\boldsymbol{\equiv}\left[ \boldsymbol{s}\left( t \right) \right]^{\boldsymbol{T}}\left( {\mathbf{E}\boldsymbol{(C}}_{m})\sqrt{{\mathbf{V}\boldsymbol{(C}}_{m})} \right):\boldsymbol{s}\left( t \right)\in S_{m}\left( \boldsymbol{P,}T \right) \right\}$$

of projections

$$\boldsymbol{s}\left( t,\boldsymbol{C}_{m} \right)\boldsymbol{\equiv}{\boldsymbol{[s}\left( t \right)]}^{\boldsymbol{T}}\left( {\mathbf{E}\boldsymbol{(C}}_{m})\sqrt{{\mathbf{V}\boldsymbol{(C}}_{m})} \right)$$

of $\boldsymbol{s}\left( t \right)\in S_{m}\left( \boldsymbol{P,}T \right)$onto the eigenspace of $\boldsymbol{C}_{n}$ is also a collection of Gaussian multivariate process with average spectrum $\boldsymbol{P}$ (where ${\mathbf{E}\boldsymbol{(C}}_{m})$,${\mathbf{V}\boldsymbol{(C}}_{m})$ are respectively the eigenvectors and diagonal matrix of eigenvalues of $\boldsymbol{C}_{m}$). The multivariate Gaussian timeseries in $S\left( \boldsymbol{P,}T,\boldsymbol{C}_{m} \right)$ exhibit the joint-spectral and covariation epochs that can arise in $m\times T$ multivariate timeseries with average spectrum $\boldsymbol{P}$ and whose full-duration covariance is brought into close approximation to $\boldsymbol{C}_{m}$ by projection onto its eigenspace. No explicit constraints are imposed on shorter-timescale epochs of covariation within elements of $S\left( \boldsymbol{P,}T,\boldsymbol{C}_{m} \right)$; the set of possibilities is shaped primarily by ancillary constraints such as $\boldsymbol{P,}T,\boldsymbol{C}_{m}$ and the linear projection by which $\boldsymbol{s}\left( t \right)$ is aligned toward $\boldsymbol{C}_{m}$.

**Wavelet-Based Metric of Spectrally-Distinguishable Temporal Epochs**

The metric $\Phi:\mathbb{R}^{T}\mathbb{\to R}$ (still under active development) of within-timeseries spectrally distinguishable temporal epochs is computed on a univariate timeseries $S(t)$, $t\in\left\{ t_{0},t_{1},t_{2},\ldots,T \right\}$ as follows (see Figure 5 in the main text):

1. Apply Matlab’s continuous wavelet transform (with the Morse wavelet) to $S(t)$. This gives an $F\times T$ matrix $W(S)$ of wavelet coefficient magnitudes. The rows of $W(S)$ are timeseries of the power in each of the $F$ frequencies. First rescale $W(S)$ by the inverse of its mean, making its elements sum to $FT$.
2. The $T\times T$ symmetric matrix $D_{Spec}(S)$ of pairwise L1 distances between the columns of $W(S)$ contains evidence of temporal variation in the core spectrum of $S$. $D_{Spec}(S)$ contains evidence of *epochal* spectral variation in $S$.
3. Finally, we set $\Phi\left( S \right)\equiv\frac{1}{FT}\mathrm{median}\left( \mathrm{uppertri}\left( D_{Spec}(S) \right) \right)$ to be the median off-diagonal values of $D_{Spec}(S)$ rescaled by the average time-frequency power in $W(S)$. Rescaling by the inverse summed power in $W(S)$ keeps the value of $\Phi$ strictly bounded in $[0,T]$.

The multivariate extension $\tilde{\Phi}$ of $\Phi$ assumes that $W(S)$ of size $F\times T$ has already been computed for some collection $\boldsymbol{S}$ of $N$ length-$T$ timeseries $S_{i}$, $i=1,2,\ldots,N$. Let $\tilde{\boldsymbol{W}}( \boldsymbol{S} )$ be the $NF\times T$ matrix of vertically concatenated $W(S_{i})$’s. Now ${\tilde{\boldsymbol{D}}}_{Spec}(\boldsymbol{S})$ is the matrix of pairwise L1 distance between columns of $\tilde{\boldsymbol{W}}( \boldsymbol{S} )$ the concatenated spectra of the $S_{i}$’s, and $\tilde{\Phi}\left( \boldsymbol{S} \right)\equiv\frac{1}{NFT}\mathrm{median}\left( \mathrm{uppertri}\left( {\tilde{\boldsymbol{D}}}_{Spec}(\boldsymbol{S}) \right) \right)$ is the appropriately rescaled median off-diagonal value of L1 distances between the time-indexed concatenated spectra of the timeseries $S_{i}$, $i=1,2,\ldots,N$.

**Performance of the Wavelet-Based Metric for Small Changes in Spectral Content**

The different univariate cases with small changes in amplitude and/or frequencies were evaluated and have been demonstrated in the figure below. Here are the details on the four toy time-series evaluated for kurtosis *(K)* and wavelet-based metric *(*$\Phi$*)*:

1. S_1_(t): Constant frequency and amplitude (used as reference);
2. S_2_(t): Constant frequency and slightly different amplitude;
3. S_3_(t): Slightly different frequency and constant amplitude; and
4. S_4_(t): Slightly different frequency and amplitude.

Higher values of the $\Phi$ metric were observed for the third and the fourth case, but not for the second case in comparison to the reference (first) case, which is understandable as the metric has been specifically designed to capture the temporal variations in the spectral content. Kurtosis did not show any significant change for any of these cases.


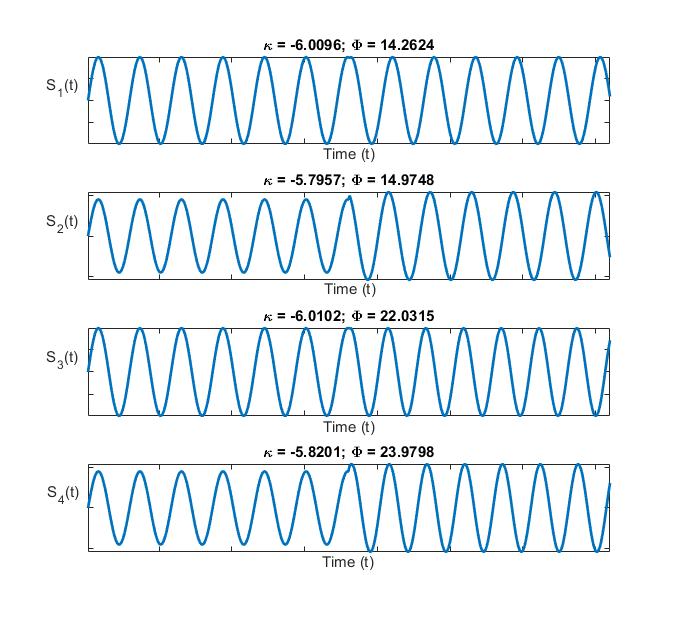


Supplementary Figure 1: Elevated values of $\Phi$ (univariate wavelet-based metric) metric were observed for small temporal variations in spectral content. Kurtosis did not show significant change in any of these different scenarios.

The multivariate version of $\Phi$ ($\tilde{\Phi}$) is computed from the median value of distances between concatenated time-indexed spectra; hence, temporal variation in spectral content of all constituent univariate time-series factors in the estimated value of this metric. Assuming epochs for all constituent univariate time-series to be varying slightly in frequency, the metric values could be expected to be lower than in cases where the temporal variation in spectral power is higher. We tested this case (slight changes in frequencies) and found that $\tilde{\Phi}$ showed higher values in these cases as compared to the stationary reference case whereas multivariate kurtosis did not show any significant change as illustrated in the figure below. The toy example in Supplementary Figure 2 estimates both metrics in three different scenarios:

1. Each constituent time-series has a constant amplitude and frequency (used as reference for comparison);
2. Each constituent time-series has three equal intervals with constant amplitude but slightly different frequencies; and
3. Each constituent time-series has three equal intervals with slightly different amplitudes and different frequencies.

Scenarios 2 and 3 both show elevated values for $\tilde{\Phi}$ but similar values for multivariate kurtosis as compared to the reference scenario 1.


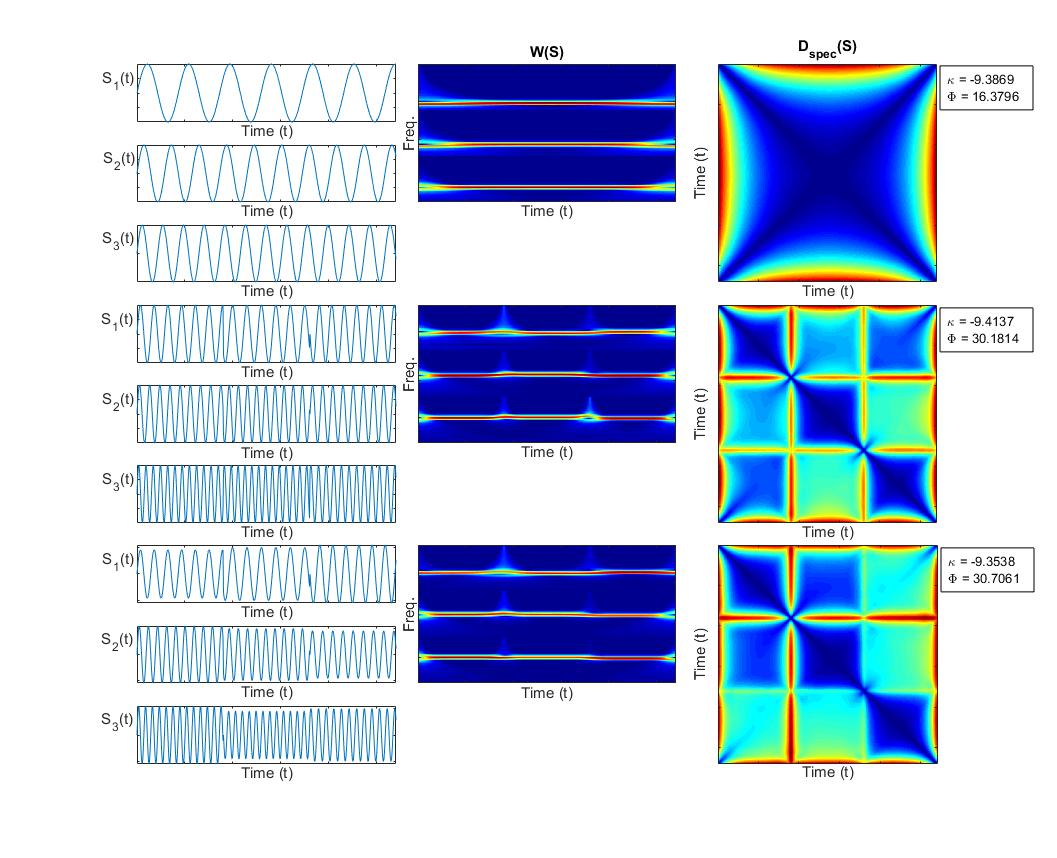


Supplementary Figure 2: Elevated values of $\tilde{\Phi}$ (multivariate wavelet-based metric) metric were observed for small temporal variations in spectral content. Similar values of multivariate kurtosis were reported in all scenarios.

**Empirical Data and Simulation Regimes**

*Real Data*

We used previously published [1] network timecourse data from a large multisite clinical resting-state fMRI study. Preprocessing and network identification followed protocols detailed in [1] that we simply outline here. Resting state functional magnetic resonance imaging data (160 volumes of echo planar imaging BOLD fMRI, TR = 2 sec.) was collected from 163 healthy controls (117 males, 46 females; mean age 36.9) and 151 age and gender matched patients with schizophrenia (114 males, 37 females; mean age 37.8) during eyes closed condition at 7 different sites across United States. After standard preprocessing, the fMRI data from all subjects was decomposed using group ICA into 100 maximally spatially independent spatial maps (http://mialab.mrn.org/software) of which 47 were identified as functionally meaningful networks. The networks fell into seven broad categories: sub-cortical (SC), auditory (AUD), visual (VIS), sensorimotor (SM), cognitive control (CC), default mode network (DMN) and cerebellar (CB). Subject specific spatial maps and timecourses were obtained from the group level spatial maps via spatio-temporal regression. The timecourses were detrended, despiked and subjected to additional postprocessing steps detailed in [1].

These timecourses (314 subjects, 47 networks, 158 timepoints), further filtered for frequencies at most $0.08$ Hz and then z-scored, are referred to below as “Real Data”. The average power at each frequency bin in $[0.003,0.08]$ Hz for all network TCs for all subjects is denoted ${\bar{\boldsymbol{P}}}_{data}$. The average cross-network covariance matrix for all subjects is denoted ${\bar{\mathbf{C}}}_{data}$.

Every simulation regime described below consists of 1000 subjects, each characterized by a set of 47, length-158 timeseries.

*SCC Gaussians*: *statistically stationary without constraint on SDTEs*

Following [2], each simulated subject in the SCC Gaussian regime is a multivariate timeseries resulting from the projection of a $47\times158$ matrix of low-pass filtered white noise spectrally matched to ${\bar{\boldsymbol{P}}}_{data}$ onto the eigenspace of ${\bar{\mathbf{C}}}_{data}$ (Figure 6 from the main text (top middle)).

*Covariance-Dynamic SCC Gaussians*: *piecewise stationary, two distinct covariance regimes, no constraint on SDTEs (CD-SCC Gaussian)*

Each simulated subject in the CD-SCC Gaussian regime starts as a set of $47$ length-$158$ low-pass filtered white noise timeseries (organized in a $47\times158$ matrix) spectrally matched to ${\bar{\boldsymbol{P}}}_{data}$. The multivariate timeseries is divided into three windows, determined by a middle window of randomly-chosen length between $40$ and $60$ TRs initially centered about the temporal midpoint $t_{m}=79$ then translated a random distance in $[0,8]$ either forward or backward. This gives a middle window (${[t}_{1},t_{2}], 40\leq\left| t_{2}-t_{1} \right|\leq60, 41\leq t_{1}\leq t_{2}\leq117$) that is roughly central but differs in extent and in degree of centrality between subjects. This simulation regime employs two target covariance matrices: one, as usual, is the mean covariance ${\bar{\mathbf{C}}}_{data}$ of the real data; the other $\boldsymbol{C}_{weak}$ is modeled on ${\bar{\mathbf{C}}}_{data}$ but with much weaker connectivity (except for preserved variances along the diagonal) and an additive layer of very low-magnitude Gaussian noise. For each subject, the random length middle window described above is projected onto the eigenspace of ${\bar{\mathbf{C}}}_{data}$, a modularly structured covariance matrix, while the first and final windows are each projected onto the eigenspace of $\boldsymbol{C}_{weak}$. The resulting multivariate timeseries are explicitly undergoing a covariance shift as they enter and leave a 40-60TR window spanning the temporal midpoint of the scan (Figure 6 from the main text (bottom left)).

*SCC Gaussians with Noise*: *statistically stationary with a single spike randomly inserted into a small proportion of network timeseries (“Noisy SCC Gaussian”)*

Each simulated subject in the Noisy SCC Gaussian regime starts as an SCC Gaussian subject (a $47\times158$ multivariate timeseries produced by subjecting white noise to spectral and covariance constraints exhibited by the real data). Of the 47 timeseries in this matrix, between 3 and 15 are selected at random to carry a single high frequency spike. This spike is a high-frequency, high-amplitude artifact centered at some randomly chosen timepoint in each of the selected networks The entire multivariate timeseries contains between 3 and 15 of these noise artifacts, with at most one appearing in any given univariate timeseries (Figure 6 from the main text (top right)).

*Spectrally and Statistically Nonstationary*: *explicitly nonstationary both statistically and epochally (“SS Nonstationary”)*

Each simulated subject in the Nonstationary regime starts as a SCC Gaussian subject (a $47\times158$ multivariate timeseries produced by subjecting white noise to spectral and covariance constraints exhibited by the real data). We designate 22 of the 47 simulated networks, (with row-indices corresponding to those of auditory, visual, sensorimotor and select cognitive control networks in $\boldsymbol{C}_{dat}$) as task-positive (TPNs). Another of 15 of the remaining 25 networks (with row-indices corresponding to default mode networks and select subcortical networks in $\boldsymbol{C}_{dat}$) as task-negative (TNNs). For each subject, a randomly selected 50%-75% of the TPNs and 50-75% of the TNNs are selected to exhibit stylized responsiveness to a hypothetical stimulus, leading each simulated subject to have at least 19 and most 29 responders among their 47 networks. Following the same procedure employed for the CD-SCC Gaussian regime, multivariate timeseries in the Nonstationary regime are divided into three windows whose endpoints are determined by a middle window of randomly-chosen length between $40$ and $60$ TRs initially centered about the temporal midpoint $t_{m}=79$ ,then translated a random distance in $[0,8]$ either forward or backward. As mentioned above, this gives a middle window (${[t}_{1},t_{2}], 40\leq\left| t_{2}-t_{1} \right|\leq60, 41\leq t_{1}\leq t_{2}\leq117$) that is roughly central but differs in extent and in degree of centrality between subjects. In those networks selected as responding TPNs, the middle window is filtered for frequency content in $[0.06,0.08]$ Hz, then rescaled to have amplitude slightly higher than the first and last windows. In responding TNNs, the middle window is filtered for frequency content in $[0.006,0.05]$ Hz and rescaled to have amplitude slightly lower than the first and last windows. So TPNs get faster and stronger in response to the hypothetical task or stimulus, while TNNs go into a slower shallower activation regime. The 19-29 nonresponding networks remain as they were following the initial spectral and covariance-matching steps. This yields a $47\times158$ matrix in which 11-17 rows contain TPN timeseries, each with a fast high-amplitude middle window, 7-11 rows contain TNN timeseries, each with slow shallow middle window and 19-29 low-pass filtered (non-windowed) SCC Gaussians unchanged after the initial spectral and covariance-matching step. This set of multivariate timeseries is explicitly spectrally and statistically nonstationary (Figure 6 from the main text (bottom middle)).

*Covariance-Dynamic Spectrally and Statistically Nonstationary*: *explicitly nonstationary both statistically and epochally with two distinct covariance regimes (“CD-SS Nonstationary”)*

Each simulated subject in the CD-Nonstationary regime starts as a Nonstationary subject as defined immediately above. However, following the CD-SCC Gaussian regime, this simulation regime employs the two, distinct target covariance matrices detailed above in the description of the CD-SCC Gaussian regime. For each subject, the random length middle window in which a subset of networks is spectrally perturbed (as detailed in the section immediately preceding) is projected onto the eigenspace of the modularly structured covariance matrix ${\bar{\mathbf{C}}}_{data}$ while the first and final windows are each projected onto the eigenspace of $\boldsymbol{C}_{weak}$. The resulting multivariate timeseries are explicitly undergoing spectral, amplitude *and* covariance shifts as they enter and leave a 40-60TR window spanning the temporal midpoint of the scan (Figure 6 from the main text (bottom right)).

**Univariate and Multivariate Kurtosis**

Univariate kurtosis, is the fourth statistical moment, $m_{4}\mathbb{=E}\left( \left( X-\mu_{X} \right)^{4} \right)$ of a random variable $X$, rescaled by the variance $m_{2}\mathbb{=E}\left( \left( X-\mu_{X} \right)^{2} \right)$ squared. For a normal random variable $\mathbb{E}\left( \frac{m_{4}}{m_{2}^{2}} \right)=3$, so *excess univariate* *kurtosis* indicative of super-Gaussianity (unusually heavy tails) is given by $b=\frac{m_{4}}{m_{2}^{2}}-3$. An unbiased estimator of excess univariate kurtosis [3] is:

|  | $\mathcal{b}_{n}=\frac{b_{n}}{{SE(b}_{n})}$ | (1) |
| --- | --- | --- |

where

|  | $b_{n}=\frac{n-1}{(n-2)(n-3)}\left( \left( n+1 \right)b+6 \right)$ | (2) |
| --- | --- | --- |

and

|  | ${SE(b}_{n})=2\sqrt{\frac{6n\left( n-1 \right)\left( n^{2}-1 \right)}{(n-2)(n+1)(n-3)(n+3)(n+5)}}$ | (3) |
| --- | --- | --- |

Multivariate kurtosis is a generalization of univariate kurtosis introduced by [4]. For a given length-$n$ multivariate process consisting of $p$ univariate timeseries, Maria’s multivariate kurtosis is defined as

|  | $B_{n,p}=\frac{1}{n}\sum_{i=1}^{n} \left( \boldsymbol{x}_{i}-\boldsymbol{\mu} \right)^{T}\boldsymbol{C}^{-1}\left( \boldsymbol{x}_{i}-\boldsymbol{\mu} \right)$ | (4) |
| --- | --- | --- |

where

|  | $C_{i,j}=\frac{1}{n}\sum_{i=1}^{n} {\left( \boldsymbol{x}_{i}-\boldsymbol{\mu} \right)\left( \boldsymbol{x}_{j}-\boldsymbol{\mu} \right)}^{T}$. | (5) |
| --- | --- | --- |

For a multivariate Gaussian process consisting of $p$ univariate timeseries of length $n\to\infty$, $\mathbb{E(}B_{n,p})=p(p+2)$. Excess multivariate kurtosis, $B_{n,p}-p(p+2)$ is evidence of multivariate super-Gaussianity. An unbiased estimator [5] of excess $B_{n,p}$ is:

|  | $\mathcal{B}_{n.p}=\frac{B_{n,p}-\frac{p\left( p+2 \right)(n-1)}{(n+1)}}{\sqrt{\frac{8p(p+2)}{n}}}.$ | (6) |
| --- | --- | --- |

$\mathcal{B}_{n.p}$has a standard normal distribution $\mathcal{N}(0,1)$ when the underlying process is actually multivariate Gaussian, so the upper tail represents strong evidence against the null hypothesis that all observations all arose from fixed Gaussian distributions in each dimension.

**Sliding Window Dynamic Connectivity**

Short-timescale network or region of interest (ROI) connectivity estimates evaluated on successive sliding windows through activation timecourses are a commonly employed [1, 6-9] vehicle through which to investigate so-called dynamic connectivity in resting-state fMRI. The general idea is straightforward: starting from a set of $R$ length-$T$ network or ROI timecourses that emerges from a standard pre-preprocessing pipeleine for rs-fMRI, slide a window of fixed length $L$ vertically through each subject’s $T\times R$ multivariate timeseries, advancing stepwise by some increment $l$ until the whole timeseries is exhausted. The window can be rectangular or have tapered edges; the pipeline that selects networks or ROIs and processes the resulting timeseries is generally unaltered relative to the non-windowed setting, and the metric of connectivity – often but not always correlation or covariance – is typically not unaltered from the non-windowed setting, in part to allow for comparisons between static (i.e., scan-length) findings and windowed (short time-scale) findings. There is considerable debate [10-13] regarding appropriate window-length subject for example, to the spectral content of the signal and other considerations. However our choice of 44s (similar to window duration as used in [1] on the same fBIRN phase 3 dataset) falls within previously recommended ranges. In background, [14] formally demonstrated use of the proposed lower limit of window length using the (inverse of minimum frequency) thumb rule as originally proposed in [12] to be overly conservative especially in moderate SNR conditions (i.e. relatively much shorter windows than as suggested by the thumb rule can be used to capture the fluctuations in time-varying connectivity). Furthermore, in their simulations, the authors in [10] indeed demonstrate that the maximum probability of detection peaks around 50 seconds. Similarly, [15] found peaks of significance of window lengths in the 40-60 seconds range. Moreover, there are several studies that corroborate that varying the window length parameter over a range beyond a certain safety limit did not change the overall observed dynamics [9, 16-19]. In summary, window size does have a substantial impact on the time-varying FC estimates as pointed out in recent papers [10-13], but recent work seems to be convergent around the 40 to 60 seconds range. Furthermore, due to the number of connectivity measurements this approach generates per subject, it is common to attempt to summarize the short-timescale connectivity patterns in the study by clustering, using $k$-means, the entire set of windowed observations, leading to some collection of $k$ summary connectivity states. These $k$ connectivity states summarizing transient connectivity patterns in the entire population yield easily computable information (e.g., occupancy rates, mean dwell times and transition probabilities) about subject-level time-varying connectivity.

As indicated above, in the section about empirical data and simulation regimes, we used previously published [1] network timecourse data from a large multisite clinical resting-state fMRI study as the empirical basis for simulation models. The original study filtered timecourses for spectral content under $[0.003, 0.125]$ Hz, but in keeping with [2], in this paper we filtered for content $[0.003, 0.08]$ Hz. Otherwise our initial pipeline leading up to windowing was identical to that published in [1] (and outlined above in the section on empirical data and simulation regimes). Again following the published study [1], our windows had length $22$ TR and were advanced by $1$ TR at each step, leading to a total of $136$ windows per subject. Although [1] used Gaussian tapering, here we employed rectangular windows. There was no discernible difference between the two approaches, so the simpler approach was utilized. Connectivity between networks on each window is measured as pairwise correlation between the windowed network timecourses. In the original study, the elbow criterion suggested $k=5$ clusters for this data, a choice that we retain here. For consistency and comparability between empirical data and the various simulation regimes, all simulation regimes were windowed and clustered (using $k=5$) with the same protocols and parameters as the empirical data.

[1] E. Damaraju, E. A. Allen, A. Belger, J. Ford, S. C. McEwen, D. Mathalon*, et al.*, "Dynamic functional connectivity analysis reveals transient states of dysconnectivity in schizophrenia," *Neuroimage: Clinical,* vol. 5, pp. 298-308, 2014.

[2] T. O. Laumann, A. Z. Snyder, A. Mitra, E. M. Gordon, C. Gratton, B. Adeyemo*, et al.*, "On the Stability of BOLD fMRI Correlations," *Cereb Cortex,* Sep 02 2016.

[3] M. K. Cain, Z. Zhang, and K. H. Yuan, "Univariate and multivariate skewness and kurtosis for measuring nonnormality: Prevalence, influence and estimation," *Behav Res Methods,* Oct 17 2016.

[4] K. V. Mardia, "Measures of Multivariate Skewness and Kurtosis with Applications," *Biometrika,* vol. 57, pp. 519-&, 1970.

[5] T. Sumikawa, K. Koizumi, and T. Seo, "Measures of multivariate skewness and kurtosis in high-dimensional framework," *Hiroshima Statistical research Group Technical Reports,* vol. 13, p. 22, 2013.

[6] B. M. Baczkowski, T. Johnstone, H. Walter, S. Erk, and I. M. Veer, "Sliding-window analysis tracks fluctuations in amygdala functional connectivity associated with physiological arousal and vigilance during fear conditioning," *Neuroimage,* vol. 153, pp. 168-178, Jun 2017.

[7] E. Premi, M. Diano, S. Gazzina, F. Cauda, S. Archetti, R. Gasparotti*, et al.*, "Dynamic connectivity state oscillations in presymptomatic GRN disease: from Connectome to Chronnectome," *Journal of Neurochemistry,* vol. 138, pp. 381-381, Aug 2016.

[8] L. Douw, D. G. Wakeman, N. Tanaka, H. S. Liu, and S. M. Stufflebeam, "State-Dependent Variability of Dynamic Functional Connectivity between Frontoparietal and Default Networks Relates to Cognitive Flexibility," *Neuroscience,* vol. 339, pp. 12-21, Dec 17 2016.

[9] E. Allen, E. Damaraju, S. M. Plis, E. Erhardt, T. Eichele, and V. D. Calhoun, "Tracking whole-brain connectivity dynamics in the resting state," *Cereb Cortex,* vol. 24, pp. 663-676, 2014.

[10] R. Hindriks, M. H. Adhikari, Y. Murayama, M. Ganzetti, D. Mantini, N. K. Logothetis*, et al.*, "Can sliding-window correlations reveal dynamic functional connectivity in resting-state fMRI?," *Neuroimage,* vol. 127, pp. 242-256, Feb 15 2016.

[11] S. Shakil, C. H. Lee, and S. D. Keilholz, "Evaluation of sliding window correlation performance for characterizing dynamic functional connectivity and brain states," *Neuroimage,* vol. 133, pp. 111-128, Jun 2016.

[12] N. Leonardi and D. Van De Ville, "On spurious and real fluctuations of dynamic functional connectivity during rest," *Neuroimage,* vol. 104, pp. 430-6, Jan 01 2015.

[13] S. Shakil, S. D. Keilholz, and L. Chin-Hui, "On frequency dependencies of sliding window correlation," in *2015 IEEE International Conference on Bioinformatics and Biomedicine (BIBM)*, 2015, pp. 363-368.

[14] A. Zalesky and M. Breakspear, "Towards a statistical test for functional connectivity dynamics," *NeuroImage,* vol. 114, pp. 466-470, 2015/07/01/ 2015.

[15] R. Liegeois, E. Ziegler, C. Phillips, P. Geurts, F. Gomez, M. A. Bahri*, et al.*, "Cerebral functional connectivity periodically (de)synchronizes with anatomical constraints," *Brain Struct Funct,* vol. 221, pp. 2985-97, Jul 2016.

[16] M. Yaesoubi, R. L. Miller, and V. D. Calhoun, "Mutually temporally independent connectivity patterns: a new framework to study the dynamics of brain connectivity at rest with application to explain group difference based on gender," *Neuroimage,* vol. 107, pp. 85-94, Feb 15 2015.

[17] X. Li, D. Zhu, X. Jiang, C. Jin, X. Zhang, L. Guo*, et al.*, "Dynamic functional connectomics signatures for characterization and differentiation of PTSD patients," *Hum Brain Mapp,* vol. 35, pp. 1761-78, Apr 2014.

[18] L. Deng, J. Sun, L. Cheng, and S. Tong, "Characterizing dynamic local functional connectivity in the human brain," *Scientific Reports,* vol. 6, p. 26976, 05/27/online 2016.

[19] M. G. Preti, T. A. W. Bolton, and D. Van De Ville, "The dynamic functional connectome: State-of-the-art and perspectives," *NeuroImage,* vol. 160, pp. 41-54, 2017/10/15/ 2017.
